# Prematurity and Low Birth Weight and Their Impact on Childhood Growth Patterns and the Risk of Long-Term Cardiovascular Sequelae

**DOI:** 10.3390/children10101599

**Published:** 2023-09-25

**Authors:** Iwona Jańczewska, Jolanta Wierzba, Alicja Jańczewska, Małgorzata Szczurek-Gierczak, Iwona Domżalska-Popadiuk

**Affiliations:** 1 Department of Neonatology, Medical University of Gdansk, Mariana Smoluchowskiego 17 Street, 80-214 Gdansk, Poland; 2 Department of Internal and Pediatric Nursing, Institute of Nursing and Midwifery, Medical University of Gdansk, Debinki 7 Street, 80-211 Gdansk, Poland; 3 Diagnostic Imaging Department, Voivodeship Oncology Centre, Skłodowskiej-Curie 2 Street, 80-210 Gdansk, Poland; 4 Department of Obstetrics and Gynecology, Pomeranian Hospitals in Gdynia, Powstania Styczniowego 1 Street, 81-519 Gdynia, Poland

**Keywords:** adults born preterm, prematurity, low birth weight, blood pressure, cardiovascular disease, growth, metabolic syndrome, obesity

## Abstract

Preterm birth (before 37 completed weeks of gestation) is a global health problem, remaining the main reason for neonatal mortality and morbidity. Improvements in perinatal and neonatal care in recent decades have been associated with a higher survival rate of extremely preterm infants, leading to a higher risk of long-term sequelae in this population throughout life. Numerous surveillance programs for formerly premature infants continue to focus on neurodevelopmental disorders, while long-term assessment of the impact of preterm birth and low birth weight on child growth and the associated risk of cardiovascular disease in young adults is equally necessary. This review will discuss the influence of prematurity and low birth weight on childhood growth and cardiovascular risk in children, adolescents and young adults. The risk of cardiovascular and metabolic disorders is increased in adult preterm survivors. In early childhood, preterm infants may show elevated blood pressure, weakened vascular growth, augmented peripheral vascular resistance and cardiomyocyte remodeling. Increased weight gain during the early postnatal period may influence later body composition, promote obesity and impair cardiovascular results. These adverse metabolic alterations contribute to an increased risk of cardiovascular incidents, adult hypertension and diabetes. Preterm-born children and those with fetal growth restriction (FGR) who demonstrate rapid changes in their weight percentile should remain under surveillance with blood pressure monitoring. A better understanding of lifelong health outcomes of preterm-born individuals is crucial for developing strategies to prevent cardiovascular sequelae and may be the basis for future research to provide effective interventions.

## 1. Introduction

Preterm birth (before 37 completed weeks of gestation) is a global health problem, remaining the main reason for neonatal mortality and morbidity [1,2].

Former preterm individuals experience subsequent long-term adverse effects of preterm birth on nutritional status, growth pattern, neurological development and heart function [3,4].

Preterm infants are a heterogeneous group. Therefore, a precise definition of this prematurity is necessary to evaluate the outcomes of this condition. The classification based on gestational age (GA) at birth includes late preterm infants (GA 34 0/7 to 36 6/7), moderately preterm birth (GA 32 0/7 to 33 6/7), very preterm birth (GA 28 0/7 to 31 6/7) and extremely preterm birth (GA < 27 6/7) [1]. Infants born preterm may be appropriate for gestational age (AGA), small for gestational age (SGA) or present fetal growth restriction (FGR). The classification by birth weight (BW) is as follows: low birth weight neonates (LBW), BW between 1500–2500 g; very low birth weight (VLBW), BW 1000–1500 g; and extremely low birth weight (ELBW), BW < 1000 g [3,4,5].

The incidence of preterm birth in developed countries has remained in the range of 11–15% over the past decades, despite advances in perinatal care [5]. Factors contributing to the persistent rate of preterm births include the use of assisted reproduction techniques, resulting in multiple and higher-risk pregnancies [6,7]. In addition, serious obstetric problems such as maternal hypertension and pre-eclampsia are more frequent, leading to preterm birth and decreased birth weight in the offspring [8]. Maternal conditions such as overweight or obesity before pregnancy can cause chronic inflammation and hormonal imbalances, and thus negatively affect the length of pregnancy [9,10,11].

Survival rates for premature infants significantly increased in the late 20th century due to the implementation of a three-level perinatal care system and the introduction of new therapies such as surfactant, antenatal corticosteroids and noninvasive ventilation into daily practice. However, prematurity still remains the cause of a number of complications underlying the increased morbidity and mortality of preterm infants, as compared with children born at term. The risk of death and the risk of complications increases with decreasing GA. Subjects born at GA less than 27 weeks had the lowest survival rate, as compared with those born at term and those born late preterm [3,4]. Early complications of prematurity include respiratory distress syndrome (RDS), intraventricular hemorrhage (IVH), necrotizing enterocolitis (NEC), sepsis, hypotension and acute renal failure. They occur in the first weeks after birth, usually have a dramatic course and contribute to the aggravation of growth failure already present at birth, which may have long-term health consequences. Late complications include neurological impairment, cerebral palsy, behavioral problems, retinopathy of prematurity (ROP) and chronic renal insufficiency [12]. Growing evidence suggests that prematurity and LBW may lead to unfavorable cardiometabolic health outcomes in young adults born preterm [13,14].

Due to advances in perinatal and neonatal care and markedly improved survival rates of extremely preterm infants in the 1990s, the number of preterm infants surviving until adolescence and adulthood continued to increase. Therefore, the long-term consequences of preterm birth, as well as the special medical needs of preterm adults, became more apparent. Numerous surveillance programs for formerly premature infants continue to focus on neurodevelopmental disorders, while long-term assessment of the impact of preterm birth and low birth weight on child growth and the associated risk of cardiovascular disease in young adults is equally necessary.

This literature review will discuss the impact of prematurity and low birth weight on childhood growth and cardiovascular risk in children, adolescents and young adults.

## 2. Methods

Papers investigating growth trajectories from infancy through adolescence to early adulthood in all categories of preterm children were taken into consideration in our review. We researched papers that assessed those parameters for groups of not only children born preterm, including extremely preterm-born infants, but also children born LBW, VLBW and ELBW. We found the recent original papers assessing the growth outcomes (weight gain, head circumference, height, BMI) extremely interesting. Papers describing the impact of long-term growth alterations on the risk of obesity and cardiovascular consequences, such as elevated blood pressure, lipid profile and the risk of death were also investigated.

We took into consideration data from large prospective cohorts assessing children born in the era of modern neonatal intensive care, as well as data derived from national medical registers.

Preterm-born children and LBW children with congenital diseases, including congenital heart diseases, were excluded from the analysis, owing to the fact that the presence of inborn anomalies affects the growth pattern negatively and is an independent risk factor for neurological sequelae and the risk factor for early death.

## 3. Start in Life: Fetal Programming

### 3.1. Early Life Programming Shapes Future Health

Preterm birth, FGR and exposure to serious complications during hospitalization in the Neonatal Intensive Care Unit (NICU) can have an essential impact on long-term health problems later in life [15]. The first 1000 days, starting from the conception have been described as “the window of opportunity for health programming”. Fetal programming means the structural and functional adaptation of developing organs to face the outside world. A concept known as the Barker hypothesis is also called the Fetal Origin of Adult Disease (FOAD) or “developmental origins of adult health and disease” (DOHAD) hypothesis [16]. According to this theory, early life exposure to unfavorable environments may lead to reprogramming of fetal structure, function and metabolism, increasing the person’s risk for cardiovascular disease later in life [14,17].

This is relevant especially to extremely preterm or ELBW children who have experienced in- and ex-utero growth restriction, which influences the risk of overweight, obesity, elevated blood pressure, stroke, heart events and metabolic syndrome in adulthood.

### 3.2. Kidney Development, Renal Impairment and the Risk for the Development of Chronic Kidney Disease (CKD)

Several studies have shown a strong epidemiological relationship between prematurity and low birth weight with adult hypertension. Preterm birth and FGR detain renal development, resulting in a decreased number of nephrons and smaller kidneys [18]. Inhibition of microvascular growth resulting in increased peripheral vascular resistance as well as dysregulation of the intrarenal renin-angiotensin system, continued oxidative stress, decreased nitric oxide production and impaired vasodilation of endothelial origin are also associated with higher blood pressure in preterm infants [14,19]. According to the Brenner hypothesis, a congenital reduction in nephron number, i.e., nephron endowment, promotes glomerular hyperfiltration, sodium and fluid retention, increased extracellular fluid (ECF) volume and elevated arterial pressure [20]. Maternal factors preceding preterm birth, including preeclampsia, low socioeconomics and neonatal acute kidney injury (AKI), also contribute to the development of hypertension and chronic kidney disease.

### 3.3. Fetal Origin of Neurologic Disorders

Long-term adaptive changes that occur in a developing fetus in response to an insecure in-utero environment affect maternal–placental–fetal (MPF) cross-talk with short- and long-term effects on brain development [21]. The results of recent cohort studies, as well as data based on national medical registries, highlight the impact of maternal health during pregnancy and maternal eating habits on the course and length of pregnancy and the size of newborns at birth. These prenatal circumstances influence the occurrence of adult-onset cerebrovascular, cognitive and neurodegenerative diseases and mental health disorders [22].

The causes of premature birth are complex. Many factors influence gene-environment interactions from conception. Various obstetric problems altering the maternal–placental–fetal (MPF) triad can negatively affect the course of pregnancy and delivery.

Origins of developmental and life course theories emphasize the importance of fetal and neonatal neurological programming on the onset of various diseases later in life. Maladaptive interactions of the MPF triad impair progenitor neuronal and glia populations in the transitional structures of the fetal brain by means of maternal immune activation, placental ischemic syndromes and fetal inflammatory reaction. Life-long developmental neuroplasticity is more likely to occur during critical periods of brain maturation within the first 1000 days. Preterm birth may also modify the programming of the hypothalamic–pituitary–adrenal axis (HPA), contributing to psychiatric disorders later in life. The HPA overactivity can also explain a cluster of features such as increased abdominal fat contents, insulin resistance, increased blood pressure, shorter adult stature and behavioral problems [23]. In most cases, neonatal neurodevelopmental care is aimed at symptomatic newborns and infants with neurological lesions, such as encephalopathy, seizures, stroke, IVH and PVL. Some patients remain asymptomatic in infancy, and neurological disorders such as developmental delay become apparent within the first 1000 days. However, these impairments are a consequence of brain diseases acquired during the prenatal and postnatal period. Early interventions, supported by health care professionals, education and parental involvement, may provide greater opportunities for recovery from these alterations. During adolescence, both former preterm patients and their caregivers, pediatricians and teachers must face neurocognitive and mental problems [21]. Thus, preterm survivors require early interventions which should be implemented during the first 1000 days of life, as well as long-term follow-up for prevention, screening and treatment of potential health consequences during the life course.

## 4. Growth Impairment

Several studies have shown that infants born preterm and with LBW may present significantly lower growth achievement in young adulthood than their peers born at term [24,25]. In the longitudinal analyses of extremely preterm and ELBW infants born in the UK in 1995, researchers reported participants’ growth trajectories by assessing their length, weight and head circumference at different age points from infancy to early adulthood. Preterm and ELBW subjects were more likely to present lower linear growth and smaller head circumference, as compared with those born at term [26].

Due to numerous complications occurring after preterm birth up to 40 weeks postmenstrual age, many preterm babies, even those who were AGA, experienced dramatic growth failure and became SGA around the term corrected age. At the expected date of delivery, the majority of these infants were smaller compared with the full-term neonates [15,24]. Despite recent progress in neonatal care, this early growth retardation appears to continue up to 3 months corrected age, affecting their subsequent long-term growth [25,27,28]. Nevertheless, compensatory catch-up growth among extremely preterm and ELBW children has been shown by many authors, but it has been observed not earlier than in infancy or childhood. The authors show different periods of this catch-up, with some studies pointing to early childhood or adolescence, while other studies show that the catch-up lasts from the age of 2 years to adulthood [15,26,29]. Participants of the EPICure study aged 2.5 to 6 years showed some catch-up in weight and height and less in head circumferences. However, at 6 years of age, these growth parameters were significantly below population norms [30]. Between 11 and 19 years of age, extremely preterm participants showed further growth catch-up, but rather in weight than in height. Therefore, the mean Body Mass Index (BMI) increased faster from 6 to 19 years of age and became greater than the population mean by 0.32 SD on average. At the age of 19 years, preterm individuals continued to be significantly shorter and lighter and presented a smaller head circumference than the control group [26]. These findings were consistent with data reported by other researchers. In a Swedish cohort of children born between 1990 and 1992, acceleration in both weight and height but not in head circumference started after 3 months of life and continued to proceed over childhood and adolescence, with a more intensive weight gain. Although the differences in growth patterns disappeared between 7 and 11 years of age, at the age of 11 years, extremely preterm participants presented shorter than their classmates in the control group [28]. A similar pattern of growth in height was observed in an Australian study of preterm infants at GA < 28 weeks who were followed up to 18 years of age. Preterm participants were shorter than full-term patients at all ages, and this disparity did not change significantly over time, while the disproportion in weight gain gradually decreased from birth to young adulthood [24]. In a Danish cohort, participants at the age of 11 to 18 years remained slightly shorter than the controls, with the most significant differences in girls. Although growth failure affected all categories of preterm infants, extremely preterm subjects, in particular, were shorter than term peers through infancy, childhood and adolescence [31,32].

Head circumference growth is the indicator of nutritional status and is independently related to long-term neurodevelopmental outcomes. Being born either very or extremely preterm and SGA contributes to abnormal head size in adolescence and young adulthood. However, it seems that lower GA at birth is associated with poorer catch-up in head circumference [26].

Studies classifying preterm participants by birth weight have shown that growth outcomes in children with FGR significantly differed from the growth pattern of full-term appropriate-weight babies. In the group of infants with FGR, after an initial marked decline in weight, height and head circumference, the rapid weight gain exceeding percentiles was reported at school age. However, they remained lighter and shorter than adults, and their final height attainment was lower than the predicted mid-parental height [33,34]. Similarly, the results of recent studies have shown that in the population of moderate and late preterm infants, differences in postnatal growth were more pronounced among SGA patients. At the age of 5 years, children SGA at birth remained shorter and lighter and showed a lower BMI compared with AGA preterm and term infants [35]. A recent study has also indicated that late preterm children born SGA could fail to recover weight and height growth during the first 36 months [36]. Moderate and late preterm infants born AGA presented catch-up growth for height, weight and BMI throughout the first year of life, leading to a decrease in disparity over time. Therefore, after 3 years of age, they had a comparable estimated mean height, weight and BMI as full-term children [32,35]. A description of selected studies assessing growth outcomes in preterm patients is presented in Table 1.

## 5. Risk of Obesity

Overweight and obesity have been identified by the World Health Organization as one of the five leading risk factors for noncommunicable disease (NCD), particularly cardiovascular disease (CVD) and diabetes mellitus [37]. In recent decades, higher mean BMI in the population and increasing prevalence of overweight and obesity have been observed globally. The incidence of obesity among children population is also rising. It has been shown that rapid weight gain and increase in BMI in preschool- and school-age individuals are risk factors for obesity and CVD in adulthood even among full-term infants [38,39]. Preterm and low birth weight infants, especially those who were SGA tend to be thinner in infancy and have lower fat stores [31]. However, the promotion of high-energy, aggressive feeding to achieve rapid catch-up in early childhood contributes to the development of obesity and abnormal fat distribution. Some studies have reported, that ELBW and SGA children who presented growth failure during infancy showed excessive weight gain in adolescence exceeding BMI percentiles, which contributed to the increased risk of insulin resistance and other cardiometabolic problems later in life [28,33,39]. A recent meta-analysis has shown that premature infants were more likely to be obese at the age of 6 to 16 years, compared with full-term infants. There was no effect of birth weight on the incidence of obesity [40]. These findings were coherent with previous studies. Rates of overweight and obesity in adulthood among extremely preterm participants were significantly higher than the population average for people at the age of 18 years. Extremely preterm participants who were obese or overweight in puberty were more likely to remain obese in adulthood [26]. Therefore, there is a growing concern that both prematurity and fetal and postnatal growth restriction, together with enhanced growth in childhood, contribute to an increased prevalence of obesity in this population, increasing the risk of developing metabolic syndrome and cardiovascular disease later in life [39,41].

## 6. Metabolic Syndrome

Metabolic syndrome is a combination of clustering risk factors, including increased waist circumference, low level of high-density lipoprotein (HDL), high triglycerides (TG), elevated blood pressure and impaired fasting glucose, which may lead to CVD and diabetes [39,42]. Some studies suggest that weight and BMI gain in ex-preterm individuals have adverse effects on subsequent fat deposition and cardiovascular health, whilst others do not show this relationship [17,43].

An initial analysis of children aged 6 years in the EPICure cohort, assessing their height and BMI at the age of 6 years, has shown that they were smaller, shorter and had a lower BMI and low fat mass. Therefore, they were not considered to have an increased risk of obesity or CVD [30]. However, completely different results were shown in the further longitudinal growth assessment of adolescents. They were proven to have an altered fat distribution at the age of 19 years, with a tendency towards visceral fat accumulation, which contributes to the metabolic syndrome and places them at a higher cardiovascular risk [41].

Several studies have reported cardiometabolic outcomes for both extremely preterm and ELBW without adjusting for the effect of size at birth on cardiometabolic health [41,44]. A Finnish cohort study has identified that a low birth weight followed by a rapid growth in weight in childhood was associated with insulin resistance and increased risk of coronary events later in life [45]. A meta-analysis of twenty-seven studies, consisting of 17,030 preterm children has found no differences between preterm and full-term subjects, regarding the majority of markers of the metabolic syndrome [46]. According to the EPICure study, GA at birth was not considered to be a good predictor for cardiometabolic risk in the extremely preterm group. Despite this, the study has found an increased frequency of the metabolic syndrome in the extremely preterm group at the age of 19 years, with those affected having a smaller size at birth. Moreover, similar to other studies, the authors have shown a positive association of excessive weight gain at pre-school age with increased BMI and systolic blood pressure in early adulthood. This relation was not influenced by size at birth [41] The adverse effects of rapid growth on metabolic characteristics have been shown in other studies. Data from an Australian cohort of extremely preterm subjects have found a link between catch-up in weight and BMI. Obesity in children aged 8 years was related to poorer glucose tolerance, poorer exercise capacity and increased visceral fat volume. These associations became stronger with age [29]. A meta-analysis by Markopoulou et al. has found strong associations between prematurity and some elements of the metabolic syndrome, such as increased total body fat mass [47]. Another meta-analysis has found a stronger correlation between LBW and the risk of metabolic syndrome, but the differences were not as significant for preterm births [48].

Controversy exists about whether parameters such as glucose and insulin levels, serum total cholesterol and low-density lipoprotein (LDL) levels and HDL cholesterol are altered in extremely preterm survivors [44,47,49]. Unsatisfactory cardiometabolic health manifested by an increase in TG level has been observed in extremely preterm participants from the Australian cohort [29]. A meta-analysis of 43 studies comparing the cardiometabolic outcomes of preterm subjects from around the world has shown elevated total cholesterol levels in preterm adults, compared with full-term adults. TG levels were also elevated, but only in European studies. In addition, the study has found higher fasting glucose and insulin levels in those born prematurely compared with those born at term. This meta-analysis has not confirmed the effect of SGA or FGR on cardiometabolic risk, considering a shorter duration of pregnancy as an independent risk factor for noncommunicable disease [47]. Late preterm participants from the Finnish ESTER cohort, beyond increased arterial blood pressure, were more likely to be obese and meet the criteria for the metabolic syndrome, compared with term individuals. Interestingly, the lipid profile was impaired only in very preterm women who presented a slight decrease in HDL levels [49]. Data from a Japanese study of LBW individuals have also found a sex-specific cardiometabolic risk. However, unlike a previous study, a high level of LDL and total cholesterol was found in LBW men, while LBW women were at risk for high blood pressure and diabetes [50]. Being of male sex was also an additional contributor to cardiometabolic risk in the Australian cohort of extremely preterm and ELBW, in which the TG level was elevated only in men, while lower HDL was reported in both male and female groups [44]. A sex-specific analysis has shown greater weight gain during childhood and adolescence in women born as LBW, compared with LBW men [47].

## 7. Hypertension

Several studies have shown a positive correlation between rapid weight gain and elevated blood pressure in extremely preterm and ELBW subjects [29,44,47,51]. Data from the Auckland steroid trial have revealed an increase in systolic blood pressure and the development of insulin resistance among a group of moderate-preterm adults compared with full-term subjects born in the 1960s–1970s [52]. Although this study had some limitations, it did not account for the presence of confounding factors such as participants’ lifestyle, an independent effect of low gestational age on the risk of cardiovascular disease seems plausible, given the recent evidence of elevated blood pressure in people born preterm.

Much of the recent evidence derives from national-based registers. Large national cohorts have the advantages of complete birth and health data and long-term follow-up [53]. Data from a Swedish cohort of more than 4 million subjects born between 1973 and 2015 have shown that preterm young adults had more antihypertensive medicines prescribed than term controls. Although the risk decreased with increasing GA, this also applied to a group of late preterm infants and was independent of the size at birth [54]. Further analysis of this population has confirmed that preterm birth was inversely related to higher hypertension risk for all groups of preterm subjects, with each additional week of pregnancy correlating with lower systolic blood pressure. In addition, data from this population have shown that the risk of other serious CVD, including cerebrovascular disease, ischemic heart disease and heart failure, across all ages examined was also increased [55,56]. This association was shown to persist from childhood to adulthood and intensified with age [57]. Elevated blood pressure was found to be independent of shared familial factors, environmental circumstances and intrauterine growth restriction. Maternal hypertension and preeclampsia were the only perinatal factors associated with elevated blood pressure and have been reported in recent studies as independent risk factors for increased both systolic and diastolic pressure in preterm offspring [49,55]. In addition, these conditions were shown as common causes of medically indicated preterm delivery, and such delivery has been reported as a stronger risk factor for hypertension and heart failure than spontaneous preterm birth [55].

Among participants from the ESTER cohort born before the 1990s, hypertension was 2- to 3-fold more frequent in ex-preterm adults, and this effect diminished with increasing GA. However, the late preterm infants also had increased both systolic and diastolic blood pressure, although not significantly [49]. Other studies and meta-analyses have confirmed a dose-response relationship between a lower GA at birth and elevated blood pressure in ex-preterm adults regardless of fetal growth disturbance [46,47,51,58]. High blood pressure is a strong risk factor for heart attack and stroke worldwide and is a common factor contributing to cardiovascular death [43].

## 8. Cardiovascular Risk

The global burden of CVD continues to increase worldwide and is responsible for 30% of all deaths. CVD includes the following disorders: ischemic heart disease, stroke, hypertensive heart disease, heart failure, cardiomyopathy and others. The known risk factors for CVD/NCD are physical inactivity, poor diet and smoking, which may lead to overweight and obesity, dyslipidemia, raised blood pressure, raised blood glucose and diabetes, i.e., components of the metabolic syndrome [59]. Newly emerging CVD risk factor includes low birth weight and prematurity.

Vascular abnormalities may be evident as an elevated pulse-wave velocity, reduced endothelium-dependent brachial artery flow-mediated dilation and increased carotid intima-media thickness [46,60]. Cardiac imaging studies have shown changes in the structure and function of the left ventricle in young adults who were born preterm, including increased left ventricular mass, right ventricular dysfunction, decreased myocardial relaxation and ejection fraction [61,62]. The hypertrophy of cardiomyocytes with reduced left ventricular diastolic function was observed during the early postnatal period. According to some studies, postnatal cardiac remodeling may also be influenced by coexisting FGR [61,62,63]. Other studies have also found a link between vascular abnormalities and FGR rather than prematurity, suggesting that FGR predisposes to changes in endothelial function [61,64]. A previous study on the VLBW cohort without distinguishing between patients born AGA and FGR, suggested that VLBW preterm-born subjects had greater vascular dysfunction and higher blood pressure, as compared with full-term adults [65]. A recent meta-analysis has found no relationship between prematurity and markers of endothelial dysfunction [47]. Although evidence from the Australian cohort of ex-preterm participants has shown that preterm birth had a relatively minor effect on the cardiovascular system with maintained micro- and macrovascular function, some derangements were observed, including reduced left ventricular and aortic size, as well as the smaller diameter of other major arteries and elevated blood pressure, which may suggest an association between impaired development of the arterial vessels system and preterm birth [66]. These findings could suggest an increased risk of stroke.

Analyses of former cohorts have reported a greater cardiovascular risk related to the length of gestation. Evaluation of preterm singletons born in Upsala in 1915–1929 has shown the connection between lower GA at birth and an increased rate of death from cerebrovascular disease, but not from ischemic heart disease. The risk of stroke diminished for patients born at GA < 36 weeks [67]. Similar outcomes were obtained in the later Swedish investigations, which included a large national cohort, reporting that preterm birth was associated with increased risks of both hemorrhagic and ischemic stroke in adulthood, but not with ischemic heart disease [68,69].

The association between prematurity and the increased risk of heart failure from childhood to adulthood has been confirmed in recent registry-based studies. The risk of heart failure increased with decreasing GA, with the highest risk being 12- and up to 17-fold greater for individuals born at GA < 28 weeks. However, late preterm subjects also had a 2.2-fold higher risk than full-term peers [57,70]. Overall, preterm birth is associated with an increased risk of death due to cardiovascular events [71]. A description of selected studies assessing cardiovascular risk in preterm patients is presented in Table 2.

## 9. Optimizing Nutrition

Optimal nutrition during the critical postnatal period is vital for the growth and development of preterm children. Early introduction of enteral nutrition reduces the risk of unfavorable consequences and improves cognitive function and metabolic outcomes later in life [72].

Nutritional practices in neonatal intensive care units have changed with advances in neonatology. In the late 20th century, it was common to feed concentrated formula to ensure adequate caloric intake, while nowadays human milk is recommended as the first choice for all newborns, including preterm infants [73,74,75]. High supplies of protein and energy during the first weeks of life are applied to improve both early growth and later neurodevelopmental outcomes. Withdrawal of this high-energy intake is recommended at 32–34 weeks postconceptional age to avoid excessive fat tissue storage and possible subsequent noncommunicable disorders. Providing a higher protein and carbohydrate diet after discharge may improve growth and body composition and have a positive impact on increased lean mass. Several studies have found a lower percentage of fat mass accumulation in exclusively human milk-fed infants compared with mainly formula-fed infants [75,76]. Thus, breastfeeding is considered to be beneficial and to reduce the risk of obesity in preterm and low birth-weight infants [36,75]. Given the evidence that promoting “excessive” catch-up growth in extremely preterm children may be associated with later cardiometabolic morbidity, the promotion of breastfeeding in this population plays a pivotal role.

Human health is notably regulated by the human genome. However, environmental non-genetic factors, such as radiation, infectious agents, chemical and environmental pollutants, lifestyle habits (e.g., tobacco, alcohol), occupation and medical interventions also play a pivotal role in regulating one’s health. This non-genetic effect complements the human genome by mediating various vital processes, such as metabolism, hormones, lipids profile, inflammation and gut microbiota modulating physical activity, oxidative stress and aging. This idea, known as the exposome concept, was first suggested by C. Wild in 2005. Nutrition is one of the environmental factors, influencing human health during the whole life course from conception onwards, contributing to the appearance of some diseases, especially noncommunicable diseases.

Environmental factors, such as inappropriate nutrition, lack of physical activity and obesity causing chronic inflammation, as well as environmental pollutants and social interactions, contribute to unfavorable cardiovascular outcomes. Scientific efforts should be applied to the development of the concept of the exposome. Advanced research on the influence of nutrition on the development of noncommunicable diseases may be helpful in determining their etiology and may contribute to the implementation of preventive methods [77,78].

## 10. Conclusions

Due to modern neonatal and pediatric care, approximately 95% of extremely preterm subjects survive until NICU discharge and beyond infancy. Therefore, the first generation of people born prematurely and with extremely low birth weight in the 1990s is now entering adulthood.

Findings from registry-based studies have reported that preterm and LBW children are at risk of growth impairment in early childhood, which may lead to obesity and, later, blood pressure alterations and other chronic cardiometabolic complications throughout childhood and adolescence to adulthood. Therefore, an early, long-term clinical follow-up for preterm-born patients is necessary for the early detection and monitoring of disorders contributing to the development of the metabolic syndrome and CVD in later life. Health practitioners should aim to reduce modifiable risk factors through dietary counseling, encouraging breastfeeding and monitoring blood pressure, glucose and lipid levels. These interventions should be supported by public health strategies and implemented from birth, through preschool and school age, and into adulthood.

Owing to the fact that elevated blood pressure appears to be strongly associated with prematurity, intensive research into the causes and effects of prematurity is needed to discover new early intervention approaches to prevent both preterm birth and prematurity-related cardiovascular sequelae.

## Figures and Tables

**Table 1:** Studies assessing growth trajectories in preterm and low birth weight individuals.

Author, Publication Year	Study/Country	Year/Study Period	N EP + Controls	Assessed Parameters	Timing of Follow-Up
Wood, N.S.; et al., 2003 [27]	GA ≤ 25 weeks EPICureUK	1995	283	height, head circumference, weight, BMI, mid-upper arm circumference	2.5 y
Ni, Y.; et al., 2020 [26]	GA ≤ 25 weeks EPICureUK	1995	129 + 65	height, head circumference, weight, BMI	11 y & 19 y
Ni, Y.; et al., 2022 [25]	EPICure GA ≤ 25 weeks EPICure2 GA < 27 weeks	1995 and 2006	EPICure n = 176; EPICure2 n = 200	height, head circumference, weight, BMI	EPICure 2.5 y, 6 y, 11 y, 19 y; EPICure2 3 y, 11 y
Farooqi, A.; et al., 2006 [28]	GA < 26 weeks Sweden; “1000-g” national Swedish cohort	1990–1992	PT 83 Controls 83	height, head circumference, weight, BMI	36 m, 11 y
Roberts, G.; et al., 2013 [24]	GA < 28 weeks Victoria, Australia	1991–1992	PT 166 Controls153	height, weight, BMI	2 y, 5 y, 8 y, 18 y
Saigal, S.; et al., 2006 [33]	ELBW < 1000 g Mean GA 27 weeks Ontario, Canada	1972–1982	PT 147 Controls145	height, weight, BMI	1 y, 2 y, 3 y, 8 y, 11–16 y, 21.5–26.5 y
Doyle, LW.; et al., 2004 [34]	ELBW < 1000 g Australia	1977–1980	ELBW 42	height, weight, BMI Relationship between patient measures and expectations at each age and relationship to growth achievement of patients’ parents.	8, 14, 20 y
Vinther, J.; et al., 2023 [32]	GA 23–43 weeks Danish National Birth Cohort	1996–2003	PT 2505 Term born 60,120	height, weight, BMI	5 m, 12 m, 7 y, 11 y, 18 y
Lindström, L.; et al., 2019 [35]	Sweden, Medical Birth Register (MBR)	2000–2015	GA 32–36 weeks n = 2224 GA > 37 weeks n = 39,445	GA at birth Weight at birth Growth trajectories in height and weight	5 y
Boyle, E.; et al., 2012 [31]	GA 32–36 weeks UK, Millenium Cohort Study	2000–2002	GA 32–36 weeks n = 1299 GA > 37 weeks n = 16,195	height, weight, BMI	3 y, 5 y

Abbreviations: BMI—Body Mass Index, ELBW—extremely low birth weight, GA—gestational age, PT—preterm, UK—the United Kingdom.

**Table 2:** Selected studies assessing the impact of prematurity and low birth weight on cardiovascular risk.

Author, Publication Year	Study Country	Year/Study Period	N EP + Controls	Assessed Parameters	Timing of Follow-Up
Bracewell, M.A.; et al., 2008 [30]	GA ≤ 25 weeks EPICureUK	1995	PT 241 Controls 160	height, head circumference, weight, BMI, BP systolic & diastolic	6 y
Ni, Y.; et al., 2021 [41]	GA ≤ 25 weeks EPICureUK		PT 129 Controls 65	height, head circumference, weight, BMI; cholesterol; (3) BP blood pressure; and (4) fasting plasma glucose3	19 y
Roberts, G.; et al., 2014 [51]	GA < 28 weeks Australia; Victorian Infant Collaborative Study	1991–1992	PT 136 Controls 120	height, weight, BMI; BP systolic & diastolic, mean	8 y, 18 y
Cheong, J.L.Y.; et al., 2023 [29]	GA < 28 weeks Australia, Victorian Infant Collaborative Study	1991–1992	PT 225 Controls 252	height, weight, BMI; body composition, glucose tolerance, lipid profiles, blood pressure, exercise capacity, BP systolic & diastolic, mean, 24 h measurement	2 y, 5 y, 8 y, 18 y, 25 y
Haikerwal, A.; et al., 2020 [57]	GA < 28 weeks ELBW < 1000 g Australia, Victorian Infant Collaborative Study	1991–1992	PT 151 Controls 119	BP systolic & diastolic, mean, 24 h measurement	18 y, 25 y
Cheong, J.L.Y.; et al., 2020 [44]	GA < 28 weeks ELBW < 1000 g Australia, Victorian Infant Collaborative Study	1991–1992	PT 165 Controls 127	Abdomen visceral fat, BP, fasting plasma glucose, lipid profile	25 y
Sipola-Leppänen, M.; et al., 2015 [49]	GA 32–34 weeks GA > 34–<37 weeks Finland ESTER cohorts	2009–2011	Early PT n = 134 Late PT n = 242 Controls 344	BMI, BP, waist circumferences, lipid profile, glucose tolerance	Mean age at assessment 23 y
Dalziel, S.R.; et al., 2007 [52]	GA 32–35 weeks New Zeland, AUCKLAND STEROID TRIAL	1964–1974	N = 458	BMI, BP, lipid profile,	30 y
Bonamy, A.; et al., 2017 [58]	GA < 27 weeks EXPRESS (Extremely Preterm Infants in Sweden Study)	2004–2007	PT 171 Controls 172	height, weight, BMI;BP, waist circumference, HR	6.5 y

Abbreviations: BMI—Body Mass Index, BP—blood pressure, ELBW—extremely low birth weight, ESTER—Preterm Birth and Early Life Programming of Adult Health and Disease (ESTER), GA—gestational age, PT—preterm, UK—the United Kingdom.

## Data Availability

Not applicable.
